# Effects of iodinated contrast agents on renal oxygenation level determined by blood oxygenation level dependent magnetic resonance imaging in rabbit models of type 1 and type 2 diabetic nephropathy

**DOI:** 10.1186/1471-2369-15-140

**Published:** 2014-09-02

**Authors:** Jia-huan Wang, Ke Ren, Wen-ge Sun, Li Zhao, Hong-shan Zhong, Ke Xu

**Affiliations:** 1 Department of Radiology, The First Hospital of China Medical University, Shenyang, Liaoning 110001, People’s Republic of China; 2 Key Laboratory of Imaging Diagnosis and Interventional Radiology of Liaoning Province, Shenyang, Liaoning 110001, People’s Republic of China

**Keywords:** Blood oxygenation level dependent magnetic resonance imaging, Diabetic nephropathy, Iodinated contrast agent, Rabbit, Pimonidazole

## Abstract

**Background:**

To evaluate the effects of contrast agents containing increasing concentrations of iodine on the renal oxygenation level determined by blood oxygenation level dependent (BOLD) magnetic resonance imaging (MRI) in a rabbit model of diabetic nephropathy.

**Methods:**

BOLD-MRI was performed using saline or iodinated (I) contrast agents (200, 240, 300, 350 and 400 mg I/mL) at 1, 24, 48, and 72 h after experimentally inducing type 2 diabetic nephropathy in rabbits. Differences in renal oxygenation levels between type 1 and type 2 diabetic nephropathy were also assessed by BOLD-MRI after injecting 400 mg I/mL of contrast agent.

**Results:**

Contrast agents increased the R2* values of the renal cortex, outer medulla, and inner medulla to the maximum levels at 24 h. The R2* values then decreased to their lowest levels at 72 h. The R2* was highest following injection of 400 mg I/mL, especially in the outer medulla. The R2* values were not significantly different between types 1 and 2 diabetic nephropathy.

**Conclusions:**

Iodinated contrast agents had the greatest influence on renal outer medulla oxygenation level at 24 h in type 2 diabetic nephropathy, with the greatest effects observed at the 400 mg I/mL dose level. There were no differences in BOLD-MRI values between type 1 and type 2 diabetic nephropathy after administering the contrast agent at 400 mg I/mL.

## Background

The development of an international economy and improvements in living standards have led to progressive increases in the morbidity and prevalence of diabetes mellitus, such that diabetes mellitus is now a major public health concern. Worldwide, 284 million people were estimated to have diabetes in 2010, representing 6.4% of the total population. Furthermore, it was predicted that 439 million individuals worldwide, 7.7% of the total population, will have diabetes mellitus in 2030 [1].

Diabetic nephropathy affects 15–25% of patients with type 1 diabetes and 30–40% of patients with type 2 diabetes [2]. Iodinated contrast agents are mainly metabolized in the kidneys. Contrast agents sometimes cause hypoxia in the kidney—a deficiency of oxygenation and the primary feature of progressive chronic kidney disease [3-5]. According to the studies by Pakfetrat et al. [6] and Toprak and Cirit [7], renal insufficiency and chronic kidney disease are major risk factors for contrast-induced nephrotoxicity (CIN), especially in patients with diabetes. CIN is defined as an increase in the serum creatinine level of ≥0.5 mg/dL or an increase of ≥25% above baseline [8]. Generally, CIN is usually transient because the serum creatinine starts to rise within 24–48 h after exposure and reaches its peak value within 3–5 days after administration of the contrast agent, and subsequently declines [9]. Therefore, early detection of CIN is challenging.

Functional magnetic resonance imaging (MRI), especially blood oxygenation level dependent (BOLD) MRI, can facilitate detailed noninvasive assessment of renal function. This technique has been used to study the effects of iodinated contrast agents on the kidney in initial feasibility studies in humans [10,11]. Tissue oxygenation bioavailability can be evaluated in terms of the R2* values. The increase of transverse relaxation rate R2* values reflect a poor oxygenation content in tissue [12]. Prasad [13] reported that the baseline R2* values in the renal medulla of spontaneously hypertensive rats were significantly greater than those of Wistar–Kyoto rats, implying lower oxygenation in spontaneously hypertensive rats. In the present study, we used BOLD-MRI to examine the effects of iodine-containing contrast agents on renal oxygenation in a New Zealand white rabbit model of diabetic nephropathy.

## Methods

### Experimental subjects

All animal procedures were approved by the ethics committee of China Medical University and complied with guidance for the Care and Use of Laboratory Animals (National Research Council, National Academy Press, Washington, DC, 1996). Ninety-six male New Zealand rabbits (6–8 months old; weighing 2.0–3.0 kg) were obtained from the Animal Department of China Medical University. All procedures were performed under general anesthesia. Food and water was withheld for 6 h during the experiments. The rabbits were allocated to three experimental groups (control, type 1 diabetic nephropathy, and type 2 diabetic nephropathy).

#### Control group

The control group comprised 12 normal rabbits that were injected with physiological saline only.

#### Type 1 diabetic nephropathy

This group included 12 rabbits, which were injected with 150 mg/kg alloxan monohydrate (Sigma Chemical Co., St, Louis, MO, USA) dissolved in physiological saline (5 g/100 mL) via a 22 G venous indwelling needle through the auricular vein. Because alloxan damages islet cells, severe hypoglycemia was expected to occur within 24–48 h after injection. Therefore, the rabbits had free access to 10% glucose water for 48 h after injection. The development of type 1 diabetes mellitus was considered successful if the blood glucose was ≥16 mmol/L [14] at 2–3 weeks after injection. The rabbits were fed a conventional diet for 12 weeks. After 12 weeks, blood glucose levels and the extent of kidney damage were assessed using blood and urine biochemical examinations.

#### Type 2 diabetic nephropathy

Type 2 diabetic nephropathy was induced in 72 rabbits by providing them a high-glucose and high-fat diet (5 g saccharose and 1 g cholesterol per 100 g of food; cholesterol was obtained from Sigma Chemical Co.). Granulated sugar with a glycemic index of >100 was dissolved in drinking water to a dilution of 1 g/mL. After 8 weeks, blood samples were obtained from the auricular vein for biochemical examinations, including fasting blood glucose, serum lipids, and high-sensitivity C reactive protein. Insulin resistance was considered to be present if these indices had increased from baseline [15]. After 12 weeks, blood glucose levels and the extent of kidney damage were assessed using blood and urine biochemical examinations.

### Preparation of contrast agents

Five iodinated (I) contrast agents were obtained from the GE Pharmaceutical Shanghai Co. Ltd. (Shanghai, China) and were used in the experiment: 200 mg I/mL (viscosity: 0.20 NS/M^2^ at 37°C; osmolality: 413 mOsm/kg H_2_O); 240 mg I/mL (viscosity: 0.33 NS/M^2^ at 37°C; osmolality: 510 mOsm/kg H_2_O); 300 mg I/mL (viscosity: 0.61 NS/M^2^ at 37°C; osmolality: 640 mOsm/kg H_2_O); 350 mg I/mL (viscosity: 1.06 NS/M^2^ at 37°C; osmolality: 780 mOsm/kg H_2_O); and 400 mg I/mL (viscosity: 1.26 NS/M^2^ at 37°C; osmolality: 920 mOsm/kg H_2_O).

### Experimental protocols

#### Protocol 1

Control rabbits and rabbits with experimentally induced type 2 diabetic nephropathy were divided into seven groups of 12 rabbits each. The control group and the positive control group (rabbits with type 2 diabetic nephropathy) were injected with physiological saline. At the same time, the other five groups of rabbits with type 2 diabetic nephropathy were injected with either 200, 240, 300, 350, or 400 mg I/mL of contrast agent (1 g iodine/kg body weight) [16]. BOLD-MRI was performed 1, 24, 48, and 72 h after injecting the contrast agent. The R2* values were determined for the renal cortex, outer medulla, and inner medulla, and were compared among the study groups at the same times.

#### Protocol 2

We selected the iodinated contrast agent that had the largest effect on the renal oxygenation level in Protocol 1 for use in Protocol 2. Rabbit models with type 1 and 2 diabetic nephropathy and control rabbits were divided into groups of 12 rabbits each. BOLD-MRI was performed 1, 24, 48, and 72 h after injecting the iodinated contrast agent. The effects of the contrast agent on the R2* values of the renal cortex, outer medulla, and inner medulla were compared among the study groups at the same times.

### MRI

#### MRI protocol

All MRI examinations were carried out using a 3.0 T Twin Speed whole-body MR scanner (General Electric Medical Systems, Milwaukee, WI, USA). A tightly fixed standard knee matrix coil with 16 independent receiver elements was used to enhance signal reception. After anesthesia, all of the rabbits were scanned in a supine position, starting at the head. The contrast agents were warmed to 37°C and were manually injected via a 22 G venous indwelling needle through the rabbit’s auricular vein. BOLD scanning was performed using a multiple-echo spoiled gradient recalled echo protocol through the center of the kidney and the following parameters, as previously described [17]: repetition time = 101.5 ms; echo time = 6.3–32 ms; flip angle = 30°; bandwidth = 31.25 kHz; matrix = 160 × 160; field of view = 18 cm × 18 cm; section thickness = 4 mm; and section number = 6.

#### Data analysis

ADVANCE 4.4 Workstation software (General Electric Medical Systems) was used to analyze the BOLD images. The R2* values were determined by placing a region of interest (ROI) marker on the upper pole, middle, and inferior pole of the kidney using coronal BOLD images. Six ROI markers were placed in the renal cortex, outer medulla, and inner medulla of each kidney and 36 ROI markers were simultaneously placed on both kidneys in each rabbit. The ROI was set to 5 mm^3^ and was placed to avoid the fatty tissues of the renal sinus and blood vessels. To prevent significant bias associated with measurement variability, each image was measured independently in triplicate by three professional radiologists, each with over 5 years’ experience.

### Pathology and immunohistochemistry

Three rabbits from each group were killed at specific time points (1, 24, 48, and 72 h). The kidneys were placed in formaldehyde solution for 24 h and then prepared for tissue dehydration, paraffin embedding, and sectioning, followed by hematoxylin and eosin staining. At a magnification of 400×, tissue injury was assessed using the following injury scoring criteria: 0–1.0, damage is limited to the glomerular structure; 1.1–2.0, damage has extended to the tubule cells; and 2.1–3.0, damage includes expansion and congestion of the capillaries.

Pimonidazole (Natural Pharmacia, Burlington, MA, USA) staining was used as an independent method to assess the extent of hypoxia in the renal tissues of the experimental groups. Renal hypoxia was evaluated semi-quantitatively based on the extent of pimonidazole staining of tubules in the cortex and medulla. Using five randomly selected images for each time point, the intensity of pimonidazole staining was assessed at a magnification of 200× according to the following criteria: 0–1.0, no staining; 1.1–2.0, moderate; and 2.1–3.0, strong staining.

Pimonidazole (60 mg/kg) was administered via intraperitoneal injection and the rabbits were killed 1.5 h later. Perfusion-fixed kidneys (3% paraformaldehyde) were cut into 4-μm-thick sections and were processed for immunostaining with an EnVision System horseradish peroxidase kit (Dako, Carpinteria, CA, USA) according to the manufacturer’s instructions. Briefly, after deparaffinization, antigen retrieval, and peroxidase quenching, the sections were incubated with protein blocking solution for 20 min. The sections were sequentially incubated with a specific rabbit anti-pimonidazole antibody diluted 1:100 (Natural Pharmacia Inc.) for 1 h, were and labeled with a polymer horseradish peroxidase-conjugated anti-rabbit secondary antibody. Pimonidazole staining was visualized using 3,3′-diaminobenzidine, followed by counterstaining with hematoxylin. Renal hypoxia was evaluated semi-quantitatively based on the extent of pimonidazole staining of tubules in the cortex and medulla.

### Statistical analysis

All data are presented as the mean ± standard deviation. Multi-level analysis of variance was used to compare the R2* values among the contrast agent-injected groups and the control group at the same time points. Rank sum tests were used to obtain non-normal distributions or heterogeneity of variance. P-values of < 0.05 were considered statistically significant. Statistical analyses were performed using SPSS software version 17.0 (SPSS Inc., Chicago, IL, USA).

## Results

The bodyweights of rabbits in all of the study groups were similar during the study (Table 1). Types 1 and 2 diabetes were successfully induced and rabbits could be differentiated based on their fasting insulin, glutamic acid decarboxylase autoantibody, and insulin autoantibody levels (Table 2). There were no differences in blood glucose, serum creatinine, blood urea nitrogen, and urinary microalbumin levels between rabbits with type 1 and type 2 diabetic nephropathy (Table 2). BOLD images were successfully acquired without dropouts and all protocol data were successfully obtained.

**Table 1:** Body weights of rabbits in each group

**Time-point**	**Control group**	**Type 1 DN group**	**Type 2 DN group**
**Baseline (kg)**	2.60 ± 0.52	2.67 ± 0.42	2.72 ± 0.51
**After 2 weeks (kg)**	2.62 ± 0.43	2.79 ± 0.43	2.89 ± 0.43
**After 12 weeks (kg)**	2.64 ± 0.48	3.12 ± 0.70	3.18 ± 0.79

*Abbreviations:DN* diabetic nephropathy.

Values are means ± standard deviation.

There were no significant differences in body weight among the three groups at any time.

**Table 2:** Blood and urine biochemical examinations at 12 weeks

	**Control group**	**Type 1 DN group**	**Type 2 DN group**
**Kidney weight (g)**	9.6 ± 1.42	10.07 ± 2.29*	10.51 ± 2.38*
**Blood glucose (mmol/L)**	4.77 ± 1.13	23.37 ± 6.72*	22.66 ± 5.00*
**SCr (μmol/L)**	56.24 ± 11.53	169.00 ± 12.08*	155.20 ± 13.27*
**BUN (mmol/L)**	4.28 ± 0.53	9.82 ± 0.76*	9.88 ± 0.69*
**Urinary microalbumin (mg/L)**	6.51 ± 1.38	21.71 ± 3.72*	21.39 ± 3.17*
**Fasting insulin (μIU/mL)**	7.61 ± 2.31	1.25 ± 1.37*	32.65 ± 2.56*
**GAD-Ab†**	−	+	−
**IAA†**	−	+	−

*Abbreviations:DN* diabetic nephropathy, *SCr* serum creatinine, *BUN* blood urea nitrogen, *GAD-Ab* glutamic acid decarboxylase autoantibody, *IAA* insulin autoantibody.

Normal ranges: blood glucose, 3.61–6.11 mmol/L; SCr, 44–133 μmol/L; BUN, 3.2–7.1 mmol/L; urinary microalbumin, <10 mg/L; fasting insulin, 5.8–18.6 μIU/mL.

Values are means ± standard deviation.

*P < 0.05 vs. control group.

There were no significant difference between types 1 and 2 diabetic nephropathy in terms of blood glucose, SCr, BUN, and urinary microalbumin results.

†Positivity for GAD-Ab and IAA is indicative of type 1 diabetes.

### Protocol 1

**Figure 1:**
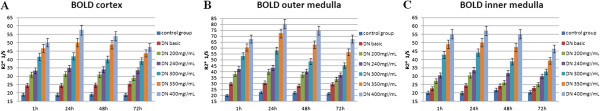
R2* values of the renal cortex (A), outer medulla (B), and inner medulla (C). Compared with the control group injected with physiological saline, the R2* values increased at 1 h and reached their peaks at 24 h and then decreased to the lowest values at 72 h in the five groups injected with iodinated contrast agents. The greatest changes occurred in the 400 mg I/mL group.

The R2* values were similar among the renal cortex, outer medulla, and inner medulla (Table 3, Figure 1). Compared with the negative control group, which was injected with physiological saline, the R2* values increased at 1 h in the experimental groups, reaching their peak values at 24 h. The R2* values then declined, reaching their lowest values at 72 h. The R2* values were consistently higher in the outer medulla than in the cortex, and increased to a peak by 24 h. Among the five dose-groups, 400 mg I/mL elicited the greatest difference in the R2* value of the outer medulla at 24 h compared with the control group (P = 0.001). These results indicate that 400 mg I/mL had the greatest effect on the renal outer medulla at 24 h. The R2* values for each part of the kidney decreased to their lowest values by 72 h. In addition, 400 mg I/mL elicited the greatest effect on the R2* value of the outer medulla at 72 h compared with the control group (P = 0.003), which indicates that there was considerable damage to the renal outer medulla at 72 h after injecting 400 mg I/mL of the contrast agent.

**Table 3:** R2* values in the control group and in the type 2 diabetic nephropathy groups injected with iodinated contrast agents

**Kidney tissue**	**Treatment**	**1 h**	**24 h**	**48 h**	**72 h**
Cortex	CG	19.0 ± 2.3	18.9 ± 2.1	19.2 ± 2.2	18.9 ± 2.7
	DN PC	24.5 ± 3.6*	24.5 ± 4.3*	24.7 ± 4.5*	25.4 ± 3.7*
	DN 200	31.1 ± 3.2*	31.3 ± 4.9*	30.9 ± 3.6*	28.9 ± 4.3*
	DN 240	33.3 ± 4.6*	34.8 ± 4.8*	34.0 ± 3.1*	33.5 ± 7.9*
	DN 300	41.7 ± 5.1*	41.9 ± 3.4*	40.1 ± 5.5*	39.2 ± 4.0*
	DN 350	46.7 ± 5.2*	50.2 ± 5.8*	48.9 ± 5.7*	43.7 ± 6.0*
	DN 400	50.1 ± 5.6*	57.6 ± 5.3*	53.9 ± 6.1*	47.4 ± 5.5*
Outer medulla	CG	20 ± 4.2	22.7 ± 4.8	22.4 ± 5.4	21.6 ± 5.1
	DN PC	29.9 ± 4.4*	30.7 ± 5.1*	28.2 ± 5.8*	29.5 ± 5.4*
	DN 200	38.1 ± 4.6*	40.1 ± 6.2*	37.5 ± 4.8*	33.8 ± 5.8*
	DN 240	42.3 ± 3.5*	43.3 ± 5.7*	40.3 ± 4.2*	37.3 ± 3.5*
	DN 300	53.4 ± 5.2*	57.9 ± 5.6*	48.8 ± 4.8*	45.4 ± 5.2*
	DN 350	60.6 ± 6.3*	72.4 ± 5.7*	62.9 ± 5.5*	56.5 ± 5.3*
	DN 400	67.6 ± 5.6*	80.4 ± 6.3*	74.7 ± 6.1*	67.7 ± 5.8*
Inner medulla	CG	20.2 ± 2.4	20.0 ± 3.6	21.8 ± 2.7	20.5 ± 3.2
	DN PC	22.7 ± 4.7*	24.5 ± 3.8*	24.3 ± 4.0*	22.9 ± 3.4*
	DN 200	27.2 ± 3.3*	30.6 ± 4.0*	26.2 ± 2.7*	25.0 ± 3.5*
	DN 240	30.4 ± 3.8*	33.6 ± 5.8*	31.9 ± 4.2*	29.9 ± 5.2*
	DN 300	42.8 ± 5.2*	44.2 ± 5.0*	38.8 ± 4.9*	32.7 ± 4.8*
	DN 350	49.2 ± 5.7*	50.2 ± 4.8*	47.3 ± 5.8*	39.4 ± 5.1*
	DN 400	55.2 ± 5.4*	57.2 ± 5.3*	55.3 ± 6.3*	46.4 ± 5.7*

*Abbreviations:CG* control group, *DN* diabetic nephropathy, *PC* positive control; *200* 200 mg I/mL contrast agent; *240* 240 mg I/mL contrast agent, *300* 300 mgI/mL contrast agent, *350* 350 mg I/mL contrast agent, *400* 400 mg I/mL contrast agent.

The control group and the DN PC group were injected with physiological saline/.

Values are means ± standard deviation.

*P < 0.05 vs. the control group.

### Protocol 2

Based on the results of Protocol 1, we used the 400 mg I/mL dose of the contrast agent in Protocol 2. Rabbits with experimentally induced type 1 or type 2 diabetic nephropathy and control rabbits underwent BOLD MRI at 1–72 h after injecting the contrast agent. The R2* values increased significantly in rabbits with diabetic nephropathy group compared with the control group. However, the R2* values were not significantly different between the two types of diabetic nephropathy in the same renal compartment at the same time point (Table 4). These results imply that the administration of 400 mg I/mL of the contrast agent had similar effects on renal oxygenation assessed by BOLD-MRI irrespective of whether diabetic nephropathy was caused by type 1 or type 2 diabetes.

**Table 4:** R2* values after an injection of 400 mg I/mL contrast agent in the control group, and type 1 and type 2 diabetic nephropathy groups

	**1 h**	**24 h**	**48 h**	**72 h**
Cortex				
CG	39.7 ± 5.4	44.2 ± 5.5	43.2 ± 4.7	37.8 ± 5.7
Type 1 DN	50.3 ± 5.5*	57.1 ± 5.2*	54.2 ± 5.9*	46.2 ± 6.5*
Type 2 DN	50.1 ± 5.6*	57.6 ± 5.3*	53.9 ± 6.1*	47.4 ± 5.5*
Outer medulla				
CG	52.7 ± 5.1	60.4 ± 6.2	53.6 ± 6.7	49.5 ± 6.2
Type 1 DN	68.6 ± 5.9*	80.3 ± 5.6*	76.7 ± 5.5*	68.1 ± 5.9*
Type 2 DN	67.6 ± 5.6*	80.4 ± 6.3*	74.7 ± 6.1*	67.7 ± 5.8*
Inner medulla				
CG	44.4 ± 5.7	49.9 ± 5.6	47.5 ± 5.6	39.9 ± 6.2
Type 1 DN	54.7 ± 6.1*	57.6 ± 5.4*	54.1 ± 6.6*	45.7 ± 6.1*
Type 2 DN	55.2 ± 5.4*	57.2 ± 5.3*	55.3 ± 6.3*	46.4 ± 5.7*

*Abbreviations:CG* control group; *DN* diabetic nephropathy.

Values are means ± standard deviation.

*P < 0.05 vs. the control group.

There were no significant differences in the R2* values between the type 1 and type 2 diabetic nephropathy groups in the same renal compartment at the same time.

### Image post-processing results

**Figure 2:**
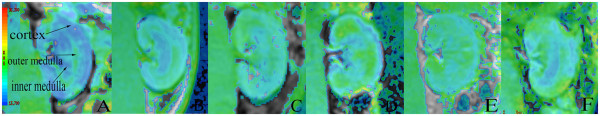
**BOLD images obtained using 400 mg I/mL of contrast agent. (A)** Control group. **(B)** Type 2 diabetic nephropathy group injected with saline. **(C–F)** Type 2 diabetic nephropathy group injected with 400 mg I/mL of contrast agent (**C**, 1 h; **D**, 24 h; **E**, 48 h; **F**, 72 h). Higher intensities on the R2* map indicate lower oxygenation of the underlying tissue. The intensity of the outer medulla of the diabetic kidney was greater than that in the cortex and inner medulla, which implies lower oxygenation of the outer medulla than the other regions.

Using the coronary BOLD image of the left kidney in a rabbit given 400 mg I/mL contrast agent as an example, it is possible to see the renal cortex, outer medulla, and inner medulla. The relative oxygenation status of the kidney could be determined based on the staining intensity on the R2* map. The area of high intensity in the outer medulla of the diabetic kidney was larger than that in the cortex and inner medulla, indicative of decreased tissue oxygenation. The BOLD images obtained at each time point are shown in Figure 2.

### Pathology and immunohistochemistry results

**Figure 3:**
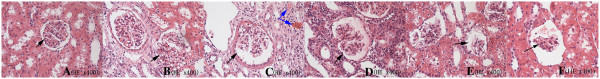
**Representative pathologic images. (A)** Control group. **(B)** Type 2 diabetic nephropathy group injected with saline. **(C–F)** Type 2 diabetic nephropathy group injected with 400 mg I/mL of contrast agent (**C**, 1 h; **D**, 24 h; **E**, 48 h; **F**, 72 h). Changes in the glomerulus, renal tubules, and interstitium can be easily seen at each time point in the type 2 diabetic nephropathy group following injection of the contrast agent .

As shown in Figure 3, using the 400 mg I/mL group of rabbits with experimentally induced type 2 diabetic nephropathy, the glomerular structure is clearly visible, without obvious atrophy shortly after injection. However, the glomeruli (black arrows) gradually atrophy over time and exhibit overt fibrosis at 72 h. The renal tubule cells become turbid and enlarged, and show cavitation changes at 24 h after contrast injection. Expansion and congestion (blue arrow) of the capillaries is also apparent and is followed by hemorrhage in the stroma. The injury scores in the negative control group and the type 2 diabetic nephropathy group without contrast injection were 0 and 0.08 ± 0.10, respectively. After injecting 400 mg I/mL contrast agent, the injury scores at 1, 24, 48, and 72 h were 0.29 ± 0.11, 0.64 ± 0.17, 0.60 ± 0.19, and 0.56 ± 0.12, respectively. Table 5 compares the injury scores between the type 1 and type 2 diabetic nephropathy groups at each time.

**Table 5:** Pathological kidney injury scores after an injection of 400 mg I/mL of contrast agent in the type 1 and type 2 diabetic nephropathy groups

	**1 h**	**24 h**	**48 h**	**72 h**
**Type 1 DN**	0.29 ± 0.15	0.63 ± 0.24	0.60 ± 0.22	0.56 ± 0.21
**Type 2 DN**	0.29 ± 0.11	0.64 ± 0.17	0.60 ± 0.19	0.56 ± 0.12

*Abbreviations:DN* diabetic nephropathy.

Values are means ± standard deviation.

There were no significant differences in injury scores between the two groups at the same times.

**Figure 4:**
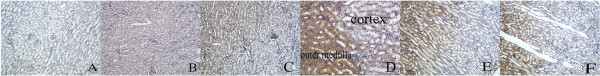
**Representative immunohistochemistry images with pimonidazole staining. (A)** Control group. **(B)** Type 2 diabetic nephropathy group injected with saline. **(C–F)** Type 2 diabetic nephropathy group injected with 400 mg I/mL of contrast agent (**C**, 1 h; **D**, 24 h; **E**, 48 h; **F**, 72 h). Pimonidazole staining was mainly localized in the renal outer medulla, especially at 24 h, while the renal cortex was lightly stained following injection of 400 mg I/mL of contrast agent.

Figure 4 shows that pimonidazole staining was mainly localized to the renal outer medulla, and the renal cortex was lightly stained. After injection of 400 mg I/mL of the contrast agent, pimonidazole staining was weaker in the cortex than in the outer medulla. The pimonidazole staining scores in the cortex and outer medulla are shown in Table 6.

**Table 6:** Pimonidazole staining scores for the renal cortex and outer medulla in rabbits injected with 400 mg I/mL of contrast agent

	**CG**	**Type 2 DN PC**	**Type 2 DN at 1 h**	**Type 2 DN at 24 h**	**Type 2 DN at 48 h**	**Type 2 DN at 72 h**
**Cortex**	0.13 ± 0.01	0.53 ± 0.05	1.35 ± 0.12*	2.17 ± 0.17*	1.93 ± 0.08*	1.77 ± 0.14*
**Outer medulla**	0.12 ± 0.01	0.51 ± 0.06	1.87 ± 0.13*	2.83 ± 0.11*	2.62 ± 0.12*	2.31 ± 0.09*

*Abbreviations:CG* control group, *DN* diabetic nephropathy, *PC* positive control.

The CG and type 2 DN PC group were injected with physiological saline.

Values are means ± standard deviation.

*P < 0.05 vs. the cortex.

Pimonidazole staining scores were significantly different between the cortex and outer medulla at 1–72 h after injecting 400 mg I/mL of contrast agent, but not in the CG or the type 2 DN PC group.

## Discussion

In this study of rabbit models of type 1 and type 2 diabetic nephropathy, we evaluated the renal blood oxygenation levels of the renal cortex, outer medulla, and inner medulla using BOLD-MRI after injecting contrast agents containing different iodine concentrations. Using non-invasive MRI, we assessed the effects of iodinated contrast agents containing different iodine concentrations on the kidneys of rabbits with experimentally induced diabetic nephropathy.

Under physiological conditions, renal oxygenation is low, especially in the outer medulla [18,19]. Palm et al. [20] reported that oxygen consumption of the renal medulla was higher in the diabetic state because of an increase in metabolic activity. Furthermore, it was reported that the kidney of diabetic rats consumed about 40% more oxygen, mainly because of the increased metabolic activity, compared with nondiabetic rats [21]. Therefore, it was concluded that hypoxia is associated with the development of diabetic nephropathy despite adequately controlled glycemia [22]. Diabetic nephropathy is associated with high oxygen consumption because of an increase in Na^+^-K^+^-ATP enzyme activity and overloading of kidney tubules, especially those in the proximal convoluted tubule and thick ascending limb located in the outer medulla [20]. These events contribute to further deteriorations in renal function [23,24]. In addition, glomerular hyperfiltration increases oxygen consumption [25]. This was confirmed by our findings that the R2* values were higher in the renal outer medulla than in the other renal compartments in the type 2 diabetic nephropathy group, which indicates that oxygen saturation is lower in the outer medulla than in other compartments.

Administration of iodinated contrast agents resulted in nephrotoxicity by increasing the permeability of glomerular cells and prompting the apoptosis of tubular cells [26]. The viscosity of iodinated contrast agent causes a reduction in arterial oxygenation [27]. Viscous contrast agents may also block small renal vessels [27]. The plasma viscosity of iodinated contrast agents is also associated with reduced renal blood flow [28]. Decrease in renal perfusion and/or increases in oxygen consumption contribute reduce the local oxygen saturation, especially in the renal outer medulla [16]. In the present study, we assessed renal oxygenation using BOLD-MRI. We found that R2* values of the renal outer medulla were higher than those of the renal cortex and inner medulla. These findings indicate that the renal outer medulla has lower oxygenation saturation and that iodinated contrast agents had greater effects on oxygenation of the renal outer medulla than on other renal components. This effect was greater in the 400 mg I/mL group than in the other groups, and was probably due to the higher iodine concentration and viscosity of the contrast agent. Consequently, the differences in R2* values might be attributable to the different iodine concentrations and the viscosities of the contrast agents. The changes in glomerular structure and the renal tubules were confirmed by pathology and the degree of hypoxia was determined by pimonidazole staining.

In our study, BOLD-MRI was performed at 1, 24, 48, and 72 h after injecting the iodinated contrast agents to observe the changes in R2* values over time. We found that the R2* values were higher after 1 h and reached peaks at 24 h after injection. The R2* values declined thereafter, reaching the lowest values at 72 h. These changes in R2* values suggest that the renal oxygenation was lowest at 24 h. Furthermore, it seems that the renal effects of the iodinated contrast agents were greatest at 24 h and gradually declined by 72 h after injection. Nevertheless, the effects of the contrast agent persisted for at least 72 h because the R2* values remained higher than that in the control group.

To study the influence of injecting iodinated contrast agents on the kidney in different types of diabetic nephropathy, we examined the effects of 400 mg I/mL contrast agent in rabbit models of type 1 and type 2 diabetic nephropathy and in control rabbits. The R2* values of both diabetic nephropathy groups were significantly greater than those of the control group. However, the R2* values were not significantly different between the type 1 and type 2 diabetic nephropathy groups. There are several possible explanations for these findings. First, although the etiologies of type 1 and type 2 diabetes differ, their effects on the kidney and the reductions on oxygenation are not significantly different [29-34]. Second, the study lasted 12 weeks, corresponding to an early stage of diabetic nephropathy. Thus, the appearance of the difference might be related to the severity of kidney damage at later stages of diabetic nephropathy.

### Limitations

First, type 1 diabetic nephropathy was established by an injection of alloxan monohydrate, and type 2 diabetic nephropathy was induced by a diet containing high levels of fat and glucose. Thus, we established models of diabetic nephropathy, which may not represent the true pathogenesis of this disease. However, it should be noted that the underlying pathogenesis of diabetic nephropathy is still poorly understood and further studies are needed in this field. Second, because the study lasted 12 weeks, it is possible that differences between type 1 and type 2 diabetic nephropathy would have been more apparent if we injected the iodinated contrast agents at a later stage of diabetic nephropathy. This possibility must be examined in future studies.

## Conclusions

This study revealed that the increases in R2* values were greatest in the renal outer medulla at 24 h after injection of iodinated contrast agents in a rabbit model of experimentally induced type 2 diabetic nephropathy. These findings indicated that renal oxygenation was lowest at 24 h after injection, particularly in the renal outer medulla. Notably, the highest dose of contrast agent tested (400 mg I/mL) had the greatest effects on the kidneys. BOLD-MRI revealed that the effects of this dose had similar effects on renal oxygenation in type 1 and type 2 diabetic nephropathy.

## Abbreviations

BOLD: Blood oxygenation level dependent; CIN: Contrast-induced nephrotoxicity; I: Iiodine; MRI: Magnetic resonance imaging; ROI: Region of interest.

## Competing interests

The authors have no conflicts of interest to declare.

## Authors’ contributions

JW, KR and HZ carried out the pathological studies and participated in the statistical analysis. JW, WS, and LZ participated in the protocol design. JW, KR and KX conceived of the study, and participated in its design and coordination and helped to draft the manuscript. All authors read and approved the final manuscript.

## Pre-publication history

The pre-publication history for this paper can be accessed here:

http://www.biomedcentral.com/1471-2369/15/140/prepub
